# Enactive Pragmatism and Ecological Psychology

**DOI:** 10.3389/fpsyg.2020.538644

**Published:** 2020-10-20

**Authors:** Matthew Crippen

**Affiliations:** ^1^Department of Philosophy, Grand Valley State University, Allendale Charter Township, MI, United States; ^2^Berlin School of Mind and Brain, Humboldt University of Berlin, Berlin, Germany

**Keywords:** affect and value, central and peripheral nervous system, ecological psychology, enactivism, gut microbiome, pragmatism and phenomenology, Physarum polycephalum, realism vs. constructivism

## Abstract

A widely cited roadblock to bridging ecological psychology and enactivism is that the former identifies with realism and the latter identifies with constructivism, which critics charge is subjectivist. A pragmatic reading, however, suggests non-mental forms of constructivism that simultaneously fit core tenets of enactivism and ecological realism. After advancing a pragmatic version of enactive constructivism that does not obviate realism, I reinforce the position with an empirical illustration: *Physarum polycephalum*, a communal unicellular organism that leaves slime trails that form chemical barriers that it avoids in foraging explorations. Here, environmental building and sensorimotor engagement are part of the same process with *P. polycephalum* coordinating around self-created, affordance-bearing geographies, which nonetheless exist independently in ways described by ecological realists. For ecological psychologists, affordances are values, meaning values are external to the perceiver. I argue that agent-enacted values have the same status and thus do not obviate ecological realism or generate subjectivism. The constructivist-realist debate organizes around the emphasis that enactivists and ecological theorists respectively place on the inner constitution of organisms vs. the structure of environments. Building on alimentary themes introduced in the *P. polycephalum* example and also in Gibson’s work, I go on to consider how environment, brain, visceral systems, and even bacteria within them enter perceptual loops. This highlights almost unfathomable degrees of mutually modulating internal and external synchronization. It also shows instances in which internal conditions alter worldly configurations and invert values, in Gibson’s sense of the term, albeit without implying subjectivism. My aim is to cut across the somatic focus of enactive constructivism and the external environment-oriented emphasis of ecological realism and show that enactivism can enrich ecological accounts of value.

## Introduction

This article starts with a commonly cited conflict: that ecological psychologists hold that environmental openings and closures for action – or affordances – remain independently of whether or not an organism is present, whereas enactivists insist that agents energetically bring forth qualities that are available to perception (see Varela et al., 1991, pp. 203–204; Fultot et al., 2016; Baggs and Chemero, 2018, 2020; Feiten, 2020; Heft, 2020; Nonaka, 2020; Segundo-Ortin, 2020). Ecological psychologists are accordingly said to favor realism and enactivists tend to lean toward quasi-idealist, constructivism.

Some commentators reject this debate as unfruitful and circumnavigate it by differentiating between environments as real affordance possibilities shared by a species and lived-worlds as constructed according to individual capacities (Baggs and Chemero, 2018, 2020). Outlooks advanced by Dewey a century ago, however, flatly suggest that constructivism need not be anti-realist in the first place. Put simply, Dewey (1920, 1925) advances a non-mental constructivism, wherein perceiving and knowing necessitates changing things or at least conditions under which they are encountered. He thereby sketches an account that retains core ideas shared by different varieties of enactivism. Simultaneously, a Deweyan rendering jettisons aspects that ecological psychologists find problematic – for example, the notion that perception arises through emergent patterns of neuronal activity (Varela et al., 1991, Ch. 8), a view not advanced by all enactivists (e.g., O’Regan and Noë, 2001). A Deweyan interpretation, moreover, offers a version of constructivism that does not obviate realism since ecological alterations, once introduced, really are there.^1^ A biological illustration is *Physarum polycephalum*: a communal unicellular organism that marks where it has been with slime secretions that it then avoids, thereby enacting or bringing forth its own geography and affordances in it. This case is typical of what enactivists cite (e.g., Thompson, 2004, Ch. 4; Noë, 2009, pp. 40–43; Di Paolo et al., 2017, Ch. 5). It is constructive insofar as *P. polycephalum* literally builds a chemical environment that immediately scaffolds its sensorimotor activity. It is simultaneously realist in senses described by ecological psychologists inasmuch as *P. polycephalum* can leave an area, with the affordance-bearing chemical barriers remaining.

For ecological psychologists, affordances are values. This means values are properties in environments, albeit defined in relation to organisms (see Gibson, 1966, p. 285, 1979, p. 127). In stripped-down form, values characterize what is favorable or hostile to an organism – a conception shared by enactivists (e.g., Thompson, 2004, Ch. 4; Colombetti, 2014, Ch. 1). Inasmuch as *P. polycephalum*’s food foraging gravitates toward unmarked and hence unexplored areas, it supplies an enactive iteration of agent-constructed values that nonetheless fits ecological definitions, which are non-subjective. Unicellular examples, however, are relatively simple, and I expect entrenched ecological psychologists to reject the *P. polycephalum* illustration as genuinely constructive, so I also examine enactive and ecological conceptions of value in cephalic creatures such as humans. The aim, once more, is to show that enactive and ecological views are not fundamentally at odds and that we need not dogmatically suppose that constructivist and realist labels obviate one another.

Though enactivists and ecological psychologists both reject representational theories, the constructivist-realist debate organizes in significant degree around the emphasis that they respectively place on the inner constitutions vs. the environments of organisms. Later portions of this article attempt to cut across this divide by examining nutritive life in cephalic creatures, articulating how visceral systems and bacteria within them alter sensorimotor activity and, by extension, values and affordances, but without diminishing their objective status. Key points advanced are (1) that gut and bacteria generated hormones and neurotransmitters alter mood, therewith environmental attunement and behavior, thus openings for action, hence perception and cognition; (2) that viscera, gut microbiota, and brain communicate reciprocally, especially around gustatory needs; (3) that gut-brain-environment activity signifies almost unfathomable degrees of mutually modulating internal and external coordination; and (4) that alimentary processes entail the detection of structure in chemical arrays inside and outside the body and, in some cases, radically change values and worlds of animals. The first two points are important to embodied cognitive science generally. The second two are specifically relevant to ecological psychology and enactivism, which are at core theories of coordination, albeit with enactivists more willing to attend to the internal milieu. Together and especially with the last point, the account cuts across body-internal and environment-external dynamics, highlighting how enactivism can enrich ecological accounts of values, while garnering a broader ecology that can accommodate both schools.

## Negotiating Constructivism and Realism

On classic renderings of enactivism, organisms “bring forth” and “enact” things rather than representing properties existing independently in the world (Dupuy and Varela, 1991; Varela, 1991; Varela et al., 1991, Chs. 8–9). Though there are different varieties of enactivism, all agree on the following: that bodily structure and objects encountered limit the way we manipulate and alter things, bringing rhythm and form to doings and undergoings and hence to the experiences arising out of them. This is not an entirely new idea but is expressed earlier by figures such as Dewey (1896) and Merleau-Ponty (1945/1962). As Dewey (1896) and enactive figures such as O’Regan and Noë (2001) reason, experience is not simply the world eliciting sensory excitations that are then wired to and interpreted by the brain. It is instead an outcome of the way sensory stimuli coordinate with motor activity and thus also around environmental contours. For this reason, perception is said to be “sensorimotor” (e.g., Dewey, 1896; Varela et al., 1991; O’Regan and Noë, 2001; Di Paolo et al., 2017); it is shaped by immediate movements and also by the history of structural coupling, along with habits, emotions, and anything else relating to actions. The key point for enactivists is that perception involves changes within local situations: “Since these local situations constantly change as a result of the perceiver’s activity, the reference point for understanding perception is no longer a pregiven, perceiver independent world but rather the sensorimotor structure of the perceiver” (Varela et al., 1991, p. 173).

Enactivists offer a range of standard illustrations, which are not obviously antagonistic to ecological psychology, but nonetheless typify non-mental or “out of the brain and head” constructivism (see Noë, 2009). One example is hands coordinating around objects to bring forth shape and texture (e.g., O’Regan and Noë, 2001, p. 945; Noë, 2004, p. 73; Myin and Degenaar, 2014, p. 91; Di Paolo et al., 2017, Ch. 3; also see Peirce, 1878; Dewey, 1920, pp. 114–115; Mead, 1938/1964, Ch. 1; Merleau-Ponty, 1945/1962, pp. 367–368). Seen enactively, pliable roughness and glassy smoothness are not in sponges or bottles alone or in brains; they are enacted by fingers sinking into knobbly pliability or caressing surfaces not biting flesh; hence, these qualities are agent-generated outcomes of interactions with surroundings. Something similar holds for the sinewy toughness that a cat’s claws realize in wood or the yielding vs. unyielding property of water that emerges depending on speed of contact. Perceived properties are accordingly not represented in creatures but instead are qualities of interactions in which organisms and things outside of them partake (cf. Dewey, 1925, p. 159). The position extends to modalities such as sight. Among other attesting examples are sensory substitution devices where head-mounted cameras stimulate skin or tongue, and people actively exploring surroundings acquire an analog of vision (e.g., Varela et al., 1991, Ch. 8; O’Regan and Noë, 2001; Noë, 2004, Ch. 2; Di Paolo et al., 2017, Ch. 5). Here, perception is not reduced to sensation since a vision-like modality can be achieved without stimulating retinal cells. Perception is instead an outcome of the manner in which sensation and motor activity coordinate around environmental contours. For such reasons, enactivists identify perception as skilled acting (e.g., O’Regan and Noë, 2001; Noë, 2004; Thompson, 2004; Di Paolo et al., 2017), repeating Dewey (1917, p. 11, 1920, p. 79, 1925, p. 330) who argued that having experience means being experienced with consciousness only incidental.^2^

By virtue of arising in this way, perception characteristically entails a gross synchronization of bodily capacities around environmental structures as when legs, feet, arms, hands, and eyes collaboratively work to keep a car on the road ahead. Through such histories of structural coupling, we develop habits or skills that allow us to perceive avenues for action, even when we happen to be sedentary (see Varela et al., 1991, Ch. 8; O’Regan and Noë, 2001; Di Paolo et al., 2017, throughout). Enactivists sometimes extend this to an evolutionary level. Varela et al. (1991), for example, have argued that bee ancestors had sensitivity to UV light and that flowers with higher reflectance in this bandwidth pollinated more successfully. Bees with more sensitivity to UV frequencies likewise gathered more food, fostering the spread of their hive’s genes. This combination of pressures led to increases in UV reflectance in flowers and sensitivity in bees. Though an uncontroversial account of coevolution, Varela et al. (1991, p. 202) cite it – somewhat contentiously – as an “example of how environmental regularities are not pregiven but are rather enacted or brought forth by a history of coupling.”

As with enactivists, ecological psychologists embrace evolution and maintain that perception occurs in a total system that includes agents and environments (see Gibson, 1966, 1979, 1992) and that we learn to perceive (Gibson, 1969; Jacobs and Michaels, 2007; Joh et al., 2007; Walter et al., 2017; Adolph et al., 2020). They are adamant that perception is not built up from sensory units akin to pixels, reinforcing the claim with Gestalt diagrams where we register entire shapes despite occluded portions (Gibson, 1979, Ch. 11; Heft, 2020). Ecological psychologists also stress the organism’s role in revealing environmental information as when discovering affordances by palpating soft objects (Gibson et al., 1987). However, they differ from enactivists in holding that perceivers do not add organization to what is received from the environment but register pre-existing structure. This means detecting affordances for action that are specified in an ambient array of energy (Gibson and Pick, 2000, pp. 15–16). An affordance, in turn, is said to be

neither an objective property nor a subjective property; or it is both… An affordance cuts across the dichotomy of subjective-objective and helps us to understand its inadequacy. It is equally a fact of the environment and a fact of behavior. It is both physical and psychical, yet neither. An affordance points both ways to the environment and to the observer (Gibson, 1979, p. 129).

At the same time, affordances “are in a sense objective, real” (Gibson, 1979, p. 129). That is, they are really in the world but are co-determined by an organism’s capacities, so that water is walkable for some insects, yet not for humans (Gibson and Pick, 2000).

Ecological psychologists thus ally themselves with realism, arguing that “since an affordance is an objective property of the environment, it exists whether or not it is perceived or realized” (Gibson and Pick, 2000, p. 16). Realism is the view that properties exist independently of agents, so that wood is smooth or sinewy regardless of whether human fingers caress it or cat claws dig into it. In the case of ecological psychology, the position is a little more nuanced since affordances are relative to organisms, yet nonetheless independent. On this view, a chair affords sitting, regardless of whether anybody is there. As Heft (2020, para. 41) writes: “It is independent of me in the respect that it is in the next room; nothing that I do from here will affect it. But it only exists as an affordance possibility relative to me (or some other person).”

Ecological psychologists are accordingly antagonistic to enactive views – for instance, the claim that “the properties that specify what colors are simply have no nonexperiential, physical counterparts” (Varela et al., 1991, p. 166). They are hostile, first, because they do not stress phenomenal sensations, and second, because they hold that perception is grounded in the physical environment. It should be added as a caveat that colors are here not understood as sensations, but as emergent phenomenal attributes of things, volumes, and so forth, though this does not dispense with the objection. Yet, the objection can be dealt with if “experience” is understood in Dewey’s sense of being a quasi-skilled interaction, which is also the view of enactivists such as O’Regan and Noë (2001). This still leaves the constructivist-realist debate that the founders of enactivism – Varela et al. (1991) – aggressively introduced and that has been steadfastly maintained by proponents on both sides. As proposed at the outset, however, Dewey offers a way out of this.

Dewey is occasionally acknowledged as a forerunner to ecological psychology (Gibson, 1982, 1988) with other pragmatists such as William James more squarely recognized (Gibson 1979, p. xiii; Heft, 2020). It is also widely accepted that Dewey anticipated enactivism (see Gallagher, 2009, 2017; Crippen, 2016, 2017; MacKenzie, 2016; Barrett, 2019). In line with this, Dewey’s (1920, 1925, 1934) work is unequivocally constructivist (see Hickman et al., 2009). Taking a cue from quantum mechanics, Dewey (1929, p. 84; also see pp. 87, 202–203) centrally argues that perceiving and knowing entails introducing changes to the world or altering conditions under which we observe it, which he regards as “the same thing in principle”. Such occurs when we thump things, hit one particle with another in quantum experiments, illuminate objects, or bend starlight with magnifying mirrors. It occurs when agents perceive and realize the properties of smoothness or sinewy toughness by caressing fingers over sanded wood or digging claws into it.

Echoing Gibson’s observation that affordances are neither subjective nor objective while simultaneously advancing a proto-enactivist view, Dewey (1934, p. 177) accordingly notes: “We speak of perception *and* its object. But perception and *its* object are built up and completed in one and the same continuing operation.” He observes more broadly that most properties are already standpoint-dependent even before agents are introduced, citing non-classical physics (see Dewey, 1929, pp. 128–129; also see Crippen, 2010, 2019a). Thus, even properties such as mass and length vary according to relative velocity, meaning they, too, are unspecifiable outside of specific points of observation. On this view, the emergence or realization of properties depends on interaction since an isolated object is potentially many different lengths, masses, textures, and so forth. One might call the position idealist since there is a sense in which properties depend on observers; only for Dewey none of this is contingent on what occurs inside the head. So, to bring the discussion down to earth, when he speaks of “social construction,” he is not referring to mental projections but to actual arrangements in the cultural fabric, good or ill, advancing a position that can be trivially read as ecological or enactive. Hence, a woman might see a setting as more threatening by virtue of it posing more objective risk to her than it does to men (Crippen and Klement, 2020). Her perception of the risk has to do with the physical arrangement of the place, but also rhythms of human movement and contact enacted in the space, which give the environment specific value relative to the woman.

The key, for Dewey, is that determinable qualities arise by changing reality or adopting a specified stance toward it, and he specifically maintains that perception emerges out of doings and effects undergone in consequence. Though not said in reference to Dewey, Chemero (2009, p. 152) nicely expresses the point when he writes that “an animal’s activities alter the world as the animal experiences it, and these alterations to the phenomenological-cognitive-behavioral niche, in turn, affect the animal’s behavior.” The view seems to be a kind of constructivism and thus to align more with enactivism than ecological psychology (see Fultot et al., 2016). However, Dewey (1925, Ch. 9) clarifies by adding that objection from the alleged side of realism that constructivism makes perception and knowledge a distortion follows simply from a confusion of tense. It is not that agents bestow upon things traits that *do not* belong to them; it is instead that activity confers characteristics that *did not* belong to things, and when bestowed, these properties are really there in the world. Seen accordingly, the constructivist-realist debate is overstated with the two positions implying practically the same thing in at least some contexts. Moreover, to the extent that properties are brought forth in the world, Dewey’s pragmatism and closely allied enactive stances do not imply subjectivism, a concern for some ecological psychologists.

This does not mean that ecological psychology and enactivism are interchangeable; they focus on overlapping but still different scales and sides of phenomenon (see Stapleton, 2016; Gastelum, 2020). Whereas enactivism, for example, explains the microstructures of immediately unfolding experience (see Varela, 1999, pp. 9–11; Kiverstein and Rietveld, 2018), affordance theory offers a good macro-level understanding of perceptual functioning; it also helps account for prospective perception (Gibson and Pick, 2000, throughout) since avenues for action are, by definition, future possibilities. Thus, if scanning a field with pear trees and wild strawberries, we register prospects for walking, climbing, and eating. Suppose we next reach for a pear, bringing it to our mouth and biting into it, our jaw and tongue coordinating around it, our saliva converting starches into sugars. Explaining how these actions integrate into experience falls more within the purview of enactivism, which has more to say about the experiential side, though ecological psychology is hardly averse to such elucidations. Gibson (1966, pp. 138–139) illustrates this when he characterizes gustatory engagements as “exploratory and stimulus-producing, since chewing releases fluids and aromas, and the movements of the tongue bring them to the chemically receptive areas. Tasting is a kind of attention, and the mouth can be said to focus on its contents.” Tasting also changes foods encountered, engendering properties.^3^ However, if this is constructive, it is simultaneously realist because new traits, once introduced, really are there. Hence, constructivism vs. realism does not appear to present an insurmountable divide between enactivism and ecological psychology and should not prevent cognitive scientists from building bridges in order to render a more complete understanding of embodied life.

## Constructing Real Worlds

Dewey (1920, p. 91) maintains that the body performs operations traditionally attributed to inner mechanisms of mind by means of “adaptive courses of action, habits, active functions, connections of doing and undergoing” and “sensori-motor co-ordinations.” Citing an amoeba as an example, he observes it must interact with its environment, else perish, and that this cannot happen any way whatever. Its capacity to move materials in and out of itself, its locomotive powers, size, shape, and things encountered in its environment all constrain and enable its behavior. Consequently, its activity has “organization,” “reference to its surroundings” and “continuity in time.” Examples like this are popular among enactivists (see Thompson, 2004, Ch. 4; Noë, 2009, pp. 40–43; Di Paolo et al., 2017, Ch. 5) with ecological psychologists also exploring unicellular life (e.g., Turvey and Carello, 2012). Examples like this, moreover, can be adapted to illustrate that constructivism does not inevitably violate realism.

*Physarum polycephalum*, a variety of slime mold, supplies a detailed case study with biologists linking its behavior to Rodney Brooks’s robotics models (Reid et al., 2012), in turn emphasized by enactivists (see Varela et al., 1991, Ch. 9). In particular, enactivists lay weight on Brooks (1999, p. 115) claim that the world – and not representations of it – is “its own best model,” and the “trick is to sense it appropriately and often enough.” With programing layers in play, and the world constraining sensory-action dynamics, intelligent patterns emerge. *P. polycephalum* responds to information in the chemical and ambient energy array and also parallels Brooks’s random wandering programs by engaging in exploratory expansion when nourishment is depleted (Latty and Beekman, 2009). Brooks’s robots have approach-avoid programs, and *P. polycephalum* achieves the same *via* chemo-attractant and chemo-aversive interactions. Binding receptors on outer membranes respond to food molecules, increasing oscillation and reducing tension in areas nearest to nutrients, provoking movement toward attractants (Ueda et al., 1980; Latty and Beekman, 2011). Upon detecting excessive salt, light, and other repellents, membrane tension increases and oscillations decrease, causing withdrawal (Ueda et al., 1980). These patterns, moreover, depend on adjustments of neighboring cells (Reid et al., 2012), meaning they are collectively brought forth and thus are proto-social. These processes also depend on molecular binding and hence introducing minor alterations to the environing chemistry.

*P. polycephalum* additionally shows capacities to anticipate periodic timing of hostile conditions (Nakagaki et al., 2000). These creatures also display remarkable foraging abilities, preferentially migrating toward optimal combinations of carbohydrates and proteins (Dussutour et al., 2010). As impressively – and this is key – they collectively navigate labyrinthine mazes and solve shortest-path problems (e.g., Nakagaki et al., 2007). One navigation mechanism is the secretion of non-living slime, which they avoid in future explorations until exhausting other alternatives. Along comparable lines, they retract cytoplasm from areas not containing nutrients, leaving tubules efficiently connecting food sources. Using these mechanisms – slime and cytoplasmic tubules – these organisms record past movements externally (Reid et al., 2012, 2013); they thereby organize their space, their local situation, and hence their sensorimotor engagements, largely according to resource availability. This means that they construct affordance-bearing chemical geographies that function as external memory traces in the vein of Clark and Chalmer’s (1998; also see Clark 2008) extended mind thesis (Crippen, 2019a).

Gibson (1966, p. 285) frames affordances as values, so his outlook would imply that *P. polycephalum*’s behavior is valuative. His book *The Senses Considered as Perceptual Systems* states that the term “affordance” was coined “as a substitute for values” to avoid subjective connotations that traditionally go with the latter. “Values” here connote “simply what things furnish, for good or ill. What they afford the observer, after all, depends on their properties.” Gibson’s (1979, p. 127) last book adds: “This is a radical hypothesis, for it implies that the “values” and “meanings” of things in the environment can be directly perceived. Moreover, it would explain the sense in which values and meanings are external to the perceiver.” Enactivists have likewise suggested that single-celled life is valuative (e.g., Thompson, 2004, Ch. 4; Thompson and Stapleton, 2008; Colombetti, 2014, Ch. 1) and for roughly the same reasons as ecological theorists. Colombetti (2014) writes: “The important point is that the sugar gradient, for the bacteria, is not just a neutral physiochemical world.” It is also “an Umwelt with a specific range of values for them: sugar is good, more sugar is better, less sugar is worse, noxious substance is bad, and so on” (p. 17; cf. Gibson, 1979, p. 140).

In sensorimotor explorations for food, which entail negotiating values and are perceptive for enactivists, *P. polycephalum* solves wayfinding problems that people would find difficult if navigating without an aerial view. So, in addition to and by virtue of being value-oriented and unambiguously sensorimotor, its behaviors are also cognitively intelligent. As importantly, a single response – for instance, foraging movements away from an area already marked as explored with slime – is all of this at once, suggesting that action, cognition, perception, and valuation fuse in even relatively simple instances of life. These creatures, then, actively shape perceptually and cognitively available, value-laden environments. They do this by laying down openings and closures for movement – in other words, affordances – which scaffold their behavior and delineate their worlds (Crippen, 2019a). These occurrences are rather unlike a beaver building a dam or other affordance structures and then perceiving them – an example that the ecological theorists Fultot et al. (2016, p. 303) deploy to undermine enactive and hence constructive accounts of perception. Specifically, they argue that “perceiving the dam, even if one wishes to characterize perception as a form of construction, is entirely different from building it.” Only in the case of *P. polycephalum*, building and what enactivists see as perceiving are entirely connected. They are entirely connected because *P. polycephalum*’s construction of slime trails simultaneously entails sensing and repulsing from them, that is, sensorimotor coordinations. The laying down of slime is therefore constitutive of sensorimotor activity, which is equivalent to perception for enactivists. Notice, however, that the constructed chemical geographies and indeed affordances retain independent existence in the same sense that furniture in an empty room does. *P. polycephalum*’s behavior is accordingly archetypically enactive and ecological at the same time and shows that constructivism need not violate realism.

While the compatibility of constructivism and realism, and accompanying lack of subjective dimensions, is straightforward in the case of *P. polycephalum*, affairs become more complicated for cephalic organisms such as humans. One factor is that values, insofar as we can tell, are more or less the same for all members of *P. polycephalum*, which is not the case for humans. A gorge might afford flying and have that value to a youthful paraglider in an energetic mood and having requisite tools and training, and something different to an exhausted octogenarian lacking appropriate skill, desire, and equipment (see Witt et al., 2005; Witt and Proffitt, 2008; Gallagher and Bower, 2014; Jensen and Pedersen, 2016). The same holds on a more temporary basis with studies suggesting that fatigue, low blood sugar, poor health, and heavy backpacks make hills look steeper or remoter because they are objectively less approachable and climbable in these circumstances (Proffitt et al., 1995; Bhalla and Proffitt, 1999; Schnall et al., 2010; Zadra et al., 2010). Positive and negative affect – corresponding to higher or lower energy and hence objective mobility – similarly alters affordances with sadness increasing perceived steepness (Riener et al., 2011). So similarly in social-political situations: citizens of an authoritarian regime may face greater danger than tourists and hence register a space such as Tahrir Square differently (see Crippen, 2019b; Crippen and Klement, 2020).

The above cited experiments and examples accordingly reiterate that affordances vary with capacities, while stressing that valuative encounters need not be subjective impressions and can instead mark real differences in ecological relations (see Gibson, 1979, pp. 134–143). They simultaneously indicate ways of more thoroughly integrating affordance theory and enactivism, particularly attempts to elaborate on the role of affectivity in perception, cognition, and action (see Colombetti, 2014; Shargel and Prinz, 2018). What is at stake in Gibson’s realist stance is his claim that affordances and values are not representations of the world, but objective properties in ecological systems (Gibson, 1979, pp. 138–140), a position that enactivism does not threaten. An illustration can be drawn from Colombetti (2014, p. 12), who cites Heidegger’s (1927/1962, p. 177) suggestion that a mood is neither subjective nor objective; it assails us and comes neither from within nor without but arises from what Heidegger calls being-in-the-world (also see Förster and Strack, 1997; Shargel and Prinz, 2018). Expressed in squarely enactive terms, mood changes how we perceive and conceive things by rearranging rhythms of action – or what might be called world grammar, understood as configurations of movement and patterns of contact that generate definition in space (see Crippen, 2010, pp. 491–492); hence, affective disposition alters our capacities and therewith the affordances and values available to us, and indeed our worlds.

For phenomenologists (e.g., Heidegger, 1927/1962; Merleau-Ponty, 1945/1962), worlds and experiences are taken to be synonymous, an idea getting close to Dewey’s (1923/1983, 1951/1981) notion of experience as culture. Keep in mind, however, that Dewey and phenomenologists typically do not understand experience as conscious awareness, but as a manner of coping that engenders different ways of perceiving and cognizing. We in fact speak of the “world” or “experience of parenthood” or “parenting culture,” and likewise of “French culture,” “the French world” or “the French experience.” Worlds, in this sense, refer to the totality of habits and comportment in surroundings that are adjusted and brought out, for example, when one switches from an academic frame to a childrearing one or as one gradually learns to enact shared French cultural practices. This points to another way in which enactivists such as Thompson (2004) and Colombetti (2014) – who are especially indebted to phenomenology – argue that organisms build their own worlds. Such occurs when depressed and lacking energy to handle things in customary ways with surroundings manifesting as less accessible. In addition to this, affectivity modifies attention, therewith the cues noticed, their parsing, and how we accordingly deal with things and change them (e.g., James, 1879; Fredrickson and Branigan, 2005; Huntsinger, 2013). Modified action adjusts focus, which loops back to modulate perception and cognition (see Dewey, 1896; Förster and Strack, 1997; Clark et al., 2015).

Comparing a happy and depressed cross-country skier possessing roughly the same skills, the latter may be less sure-footed because of mood-related fatigue that in fact shows up partly in consequence of changed bodily disposition. The depressed skier may, therefore, perceive an icy hill as steeper and more forbidding because it in fact poses more risk to the weary (see Crippen, 2018). The threatening nature of the hill is again brought forth partly by the skier attacking it with greater hesitancy, not poling hard to build speed, falling into slower rhythms of doing and undergoing, perhaps plowing the snow to the side. The skier may, thereby, actualize the hill differently than the happier companion, enacting different environmental and bodily alterations, hence bringing forth different properties of snow and generating a different overall experience.

From Dewey’s standpoint – and I think from any standpoint – none of this obviates realism even while some of it is constructive. However, the mood-based behavioral dispositions do push the happy and depressed skier into somewhat different worlds to the extent that they have different capacities and thus face varying constraints. We can imagine, therefore, that the two perceive and value their worlds differently, but this is because they enact and hence find themselves in objectively different situations. So the differences are not merely in their heads. There is an additional reason that the enactive position articulated here does not entail subjectivism: because the skiers, in spite of their mood-based enactments, are still embodied similarly, retaining many of the same needs and capacities, which cultivate overwhelmingly similar environmental enactments, experiences, and indeed affordances and values. Without any complicated philosophical maneuvers or denying individual difference, we can therefore conclude that the two skiers remain in predominantly shared worlds with the same objective goods and ills.

## Broadening Ecologies

The last section examined agent-engendered affordances and values with the discussion of *P. polycephalum* focusing on active structuring of the external chemical array. Gibson’s (1966, Ch. 8) accounts of animal life attend to the chemical array, discussing food values and the difficulty detecting them. In cephalic organisms, alimentary activity involves almost inestimable levels of mutually modulating internal and external synchronization, oriented around exploiting environmental resources in order to maintain homeostasis. Gibson (1966, pp. 141–142) accordingly stresses the importance of detecting structure of the chemical array inside the body, in addition to registering it externally. Thus, Gibson himself has laid groundwork for incorporating the internal milieu into ecological psychology. This opens additional linkages between the environment-oriented emphasis of ecological realism and the more somatic-engendered thrust of enactive constructivism. For example, microbes introduced to the alimentary system can invert what Gibson calls positive and negative affordances, understood as resource openings, such as food or escape paths, and closures, such as dangerous cliffs or predators (Gibson, 1966, p. 146, 1979, pp. 137, 157, 233). These shifts entail changes in habitual handlings and hence worlds configurations, defined again as rhythms of movement and contacts enacted that fundamentally alter – or, one might say, reconstruct – the situations in which organisms find themselves. This does not threaten ecological psychology, but it arguably makes room for the inclusion of enactive ideas. It also goes some way cutting across the environmental-external and body-internal emphases of the two schools.

Note, by way of introduction, that appetitive models of psychic life are longstanding and they are fundamentally valuative (e.g., Spinoza, 1677/1996, p. 73; Aristotle, 1941; Simon, 1967; Miller, 1983; Loewenstein, 1994). Everyday language suggests awareness of this, as Johnson (2017, p. 162) notes, cataloguing numerous examples: we thirst and have insatiable appetite for knowledge; we chew the fat, and swallow proposals; someone shits out a bad, rotten, and unsavory idea – it smells fishy, leaves a foul taste; certain notions are warmed over, sugar coated, made palatable, fed to us, and forced down our throats; politicians cook up half-baked facts that we take with a grain of salt; professors digest meaty issues; students sink their teeth into food for thought, occasionally watering it down, regurgitating and spitting it back; a sleek sports car is sweet; poor décor makes us want to puke; colloquial Egyptian Arabic calls good-looking people “tasty.”

The sheer wealth of gut feelings, thoughts, and percepts is not unexpected given gustation is central to animal life and also because the gastrointestinal system is innervated in degree that some call it the “second brain” (Gershon, 1998). Consistent with this, the gut communicates reciprocally with the brain and functions as an internal sensory system. This last role is biologically vital insofar as human intestines have an internal surface area roughly 100 times the size of the skin and interface with vast ecologies containing roughly 100 trillion microorganisms from 40,000 species (Mayer, 2011). There is accordingly a great deal to handle and sometimes defend against. This is more so since gut problems have body-wide ramifications with bacteria imbalances predicting conditions, such as anxiety, depression, autism, schizophrenia, Alzheimer’s, and eating disorders (Burrus, 2012; Foster and McVey Neufeld, 2013; Severance et al., 2016; van de Wouw et al., 2017; Wong et al., 2017; Cussotto et al., 2018). Internal bacteria thus have obvious impacts on cognition, perception, and mood, and therefore dealings with the world.

Gibson (1966, p. 146) recognizes the centrality of gustation – and by extension, the gut – in animal life. He observes: “Predatory animals should come to be sensitive to the odor that specifies their prey… The cat smells the mouse. Reciprocally, the preyed-upon animal needs to be sensitive to the odor that specifies a predator.” He stresses that “this should develop early, since an error of discrimination is fatal and cannot be corrected. The mouse smells the cat,” and “the affordance of prey odor is different from that of predator odor, the one being positive the other negative.” Gibson (1979, p. 137) adds that “all these benefits and injuries, these safeties and dangers, these positive and negative affordances are properties of things taken with reference to an observer.” However, they are “not properties of the experiences of the observer. They are not subjective values; they are not feelings of pleasure or pain added to neutral perceptions.” Summing up, Gibson (1979, p. 233) writes: “The positive and negative affordances of things in the environment are what makes locomotion through the medium such a fundamental kind of behavior for animals.”

There are times, however, when internal ecologies push outward, inverting positive and negative affordances and values with frightening results, as in the case of *Toxoplasma gondii*. Infecting the brain of mice and rats after ingestion, this parasite cultivates an attraction or at least indifference to cat smells, especially urine (Berdoy et al., 2000; Vyas et al., 2007a, b; Lamberton et al., 2008; Kannan et al., 2010; Ingram et al., 2013). Reduced wayfinding capacity and a tendency to stay in the open are other symptoms (Hodková et al., 2007a; Webster, 2007). These changed manners of coordinating with the structure of the chemical and optic array increase vulnerability to predation, which serves the pathogen since it reproduces in cats to be redistributed to rodents through feces. In effect, *T. gondii* rebuilds the worlds of rodents in order to serve its biological imperatives. One might say, therefore, that infected animals become prey to *T. gondii* and, in Gibson’s (1979, p. 97) terms, act according to its values or utilities. Though typically asymptomatic in humans, infected males find cat urine more pleasant (Flegr et al., 2011); they have elevated testosterone, higher aggression, and degraded motor-control, which has the side effect of increasing car accidents (e.g., Havlíček et al., 2001; Flegr et al., 2002, 2008, 2009; Hodková et al., 2007b; Kocazeybek et al., 2009; Coccaro et al., 2016). Testosterone links to mood and thus action – or in Heidegger’s (1927/1962) phraseology, to altered being-in-the-world, which has also been characterized in terms of situation-defining habit deployments and practical handlings. In particular, it brings out a more dangerous world by motivating risky behavior, to some extent inverting what would normally be negative affordances. This may have served objective values of the pathogen in the evolutionary past since it can reproduce in large felines such as lions (Ferreira et al., 2019).

Gut bacteria similarly modulate external dispositions with probiotic interventions highlighting some causal mechanisms by which this occurs. Treatments can increase the neurotransmitter GABA along with serotonin precursors (see Wallace and Milev, 2017). GABA suppresses immunological inflammations, and ingesting it decreases fatigue and improves cognitive performance, even though it probably does not reach the brain (Kanehira et al., 2011). Conversely, inflammation and diminished serotonin link to impaired cognition and depression (see Jenkins et al., 2016) with gut bacteria also regulating glucose and energy (Gérard and Vidal, 2019), all of this relevant to active stances. Earlier-cited experiments suggested that energy levels affect affordance availability, and there is some fairly direct evidence that gut bacteria do the same and for similar reasons. Studies find that *Bifidobacteria* species alter communication in GABA receptors and decreases blood cortisol (Cryan and Dinan, 2012). Energy consumption involves both GABA and cortisol (Nieuwenhuizen and Rutters, 2008; Xu and Tong, 2011). The latter associates with stress, hence mood, habitual dispositions, and perception. Cortisol enhances negative valence in visual perception (Brown et al., 2017). Registering negative valance in objects can in turn make them appear farther away (e.g., Beloff and Beloff, 1961; Balcetis, 2016), which means less approachable.

These outcomes are not just a result of chemical diffusion from the gastrointestinal tract. They also follow from direct communication between gut and brain as demonstrated by the fact that probiotic benefits attenuate in mice with severed vagus nerves, the primary neural pathway between brain and viscera (Cryan and Dinan, 2012). The solitary nucleus – a brainstem area – is a major junction in gut-brain pathways, intercommunicating with stomach, kidneys, heart, and more (Critchley and Harrison, 2013). Involved neurons project into other subcortical regions, such as the hypothalamus and amygdala bulbs, together contributing to “coordinated autonomic, hormonal, and even immune outputs” collectively oriented toward “functional goals” (Critchley and Harrison, 2013, p. 625). These structures in turn interact with other neural regions, including cortical ones, and the brain reciprocally with the rest of the body and indeed the world. This means that in addition to sensing the internal milieu and helping synchronize it, peripheral organs cultivate coordination with the external environment, in some sense also monitoring it. Gustation is dominant in this, entering sensorimotor loops with the gut and other visceral organs supplying information about fluid balance and energy levels, modulating environmental searching accordingly (Oliveira-Maia et al., 2011; Thornton and Norgren, 2016). In response to pathogen threats, peripheral organs not only respond to problems; they indicate them, perhaps increasing gastrointestinal dysrhythmia, pulse, blood pressure, perspiration, and otherwise supplying information about internal conditions (see Horn, 2008). In conjunction with the brain and environmental contact, this helps organisms regulate action, attention, cognition, emotion, homeostasis, reward, and memory (Humphries et al., 2007; Farr et al., 2016), and therewith external information foraging (see Miller, 1983; Pirolli and Card, 1999). Shifts may be specific as when an aversion closes an illness-inducing food as a viable affordance, leaving alternatives more attractive. Conversely, studies find that thirst and nicotine deprivation make cups appear taller and cigarettes longer, and presumably more central in perception as their value increases (Brendl et al., 2003; Veltkamp et al., 2008; also see Gibson, 1979, pp. 131–134).

Gibson observes that “animals need to perceive the affordances of substances, their chemical values or utilities” (Gibson, 1979, p. 97). He further remarks that “food values of natural substances in the environment are extremely difficult to detect” (Gibson, 1966, p. 141), which is perhaps why so many systems in the body orient toward this task. Water and salt are other chemical values around which activity organizes, and the last serves as an illustration that recapitulates several central points. To begin with, brainstem regions and chorda tympani nerves, which relay taste bud information, fire proportionately to saltiness, all else equal (Thornton and Norgren, 2016). Yet, affairs are rarely equal and firing rates are lower in sodium-deprived animals (Garcia et al., 2008; Huang and Yan, 2008). This makes things taste less salty, thereby increasing sodium foraging and consumption with human subjects finding heavily salted foods less intense and more pleasant (Bertino et al., 1981). Saltiness is accordingly not a “sensory given” (Parrott and Schulkin, 1993), registered independently of homeostatic needs, in line with enactive claims. Hence, while salt is present or absent independently of an organism, the value afforded by salty foods and their resonance in perceptual systems depends on internal sodium balance. Moreover, following the logic of the earlier mentioned studies on perceived glass and cigarette size, one can speculate that salt resources increasingly stand out as they become more objectively required. This again suggests that affordances vary with need, but without making them merely subjective. It is also to propose along enactive lines that the internal milieu is part of the sensorimotor loop and involved in bringing forth environmental dimensions that are essential to cellular life.

One general lesson implied in all this is that the visceral-neural axis is environmentally situated. Something similar holds in cases of microbe-gut-brain interactions. Gut bacteria – weighing between 1 and 2 kg – are in effect an organ functioning to digest, nourish, and produce critical hormones and neurotransmitters; cephalic responses lead to the secretion of nutrients to feed bacteria, again as if they are organs in the system; bacteria in turn differentially regulate reward chemicals like dopamine, adjusting value attunement according to what and how much we ingest; bacteria further appear to influence when food is consumed, consequently shaping circadian rhythms and therewith energy levels (see Fetissov, 2017; van de Wouw et al., 2017), which has obvious implications for affordance theory and enactivism. This partly occurs through bacteria producing short chain fatty acids and hormones that regulate host appetite and metabolism (van de Wouw et al., 2017). Moreover, all this appears to occur through a dynamic looping effect with bacteria. That is, dietary choices affect gut bacteria, and gut bacteria affect dietary choices (Cussotto et al., 2018). Outcomes can be quite profound. For example, experiments with *Drosophila* – a fly species – show that altered gut microbiota lead to significant shifts in chemosensory foraging, largely olfactory guided in this case (Wong et al., 2017). Researchers speculate something similar holds for humans (Norris et al., 2013; van de Wouw et al., 2017). In Gibsonian terms, gut bacteria appear to moderate the value that chemicals in the environment have for organisms; understood enactively, gut bacteria transform behaviors and thus the animal’s world. These positions seem entirely compatible.

The upshot is that internal living ecologies interacting with their surroundings get animals to do the same through total body coordinations with the world. This means that the gut and bacteria in it – again considered as an organ – are part of sensorimotor loops. Thus, Merleau-Ponty (1945/1962, p. 272) – a biologically informed influencer of both Gibson and enactivists – was more right than he knew when he observed: the “body is not a collection of adjacent organs, but a synergic system, all the functions of which are exercised and linked together in the general action of being in the world.” Along lines of both enactivism and ecological psychology, this suggests that psychic life depends on global synchronization of capacities, only not all directed toward the external world. There is a great deal of internal regulation, albeit synchronized in large degree with the animal’s world – synchronized because external coordination calibrates the internal milieu, whether through gustation or stressful events affecting the microbiome (Cussotto et al., 2018). Gut bacteria in turn moderate feeding behavior (Fetissov, 2017; van de Wouw et al., 2017), hence environments and values in them. Specific balances appear to further coordinate external activity by increasing or decreasing stress-like behavior and related hormones (Cussotto et al., 2018). This is consistent with the enactive and phenomenological thesis that actions bring forth worlds and certain valuative tones. Insofar as this occurs partly through altered habitual tendencies, it also implies shifts in environmental affordances.

## Conclusion

I began this article by considering a widely discussed bone of contention between ecological psychology and enactivism: that the former adopts a realist position and the latter adopts a constructivist one. Antagonists from both sides frame this as a serious source of conflict. Relatedly, while both schools focus on embodied environmental life, enactivists lay comparatively more weight on agent-driven somatic structuring of perception and cognition. This is compared to ecological psychologists, who start with the environment, arguing that information from it is sufficient to structure perception. These different starting points lead enactivists to accuse ecological psychologists of neglecting individual contributions to psychic life and ecological psychologists to censure enactivists for promoting subjectivism and getting dangerously close to solipsism.

Drawing on Dewey’s analysis and a range of supporting examples, I attempted to show that there are cases in which constructivism does not obviate realism or generate subjectivism. I reinforced the claim by examining the behavior of *P. polycephalum*. Specifically, I made the case that its sensorimotor coordinations are enactments of affordances constructed in slime, adding that these outcomes are achieved without violating realist tenets. Later on, I repeated variations of this argument in an effort to show that values can be agent-enacted yet real. The last section of this article took a cue from Gibson, who emphasizes the importance of detecting the structure of chemical arrays inside the body. I expanded on the observation by sketching an ecology of the internal milieu and its relation to the external environment. The aim here was to bridge the agent-driven thrust of enactive constructivism and the environment-external orientation of ecological psychology.

The kinds of cases emphasized in this article are not at odds with ecological psychology and in fact cited in the literature, including the pioneering work of Eleanor and James Gibson. However, they get less attention in ecological quarters, which disproportionately focuses on visual perception, notwithstanding many exceptions. The object handling examples offered in this paper are ones that enactivists use to argue that we immediately “bring forth” qualities and experiences, even if one wants to object that the illustrations are unoriginal since they reoccur in pragmatism, phenomenology, and indeed ecological psychology. The chemical engagements of *P. polycephalum* and rodents infected with *T. gondii* demonstrate comparable points. Ecological psychologists, of course, talk at length about haptic and chemical perception, and they do not deny the obvious: that organisms change their environments. However, it may be that their emphasis on visual perception – which is a modality of distance (see Dewey, 1934, pp. 236–237; Gibson, 1979, p. 233) – leads them to understate the frequency and extent to which perception requires changing local surroundings. The latter suggests constructive dimensions, even while not undercutting realism.

Though I hope my case has been compelling, I would not want to argue for the universal status of conclusions I have drawn, and this is part of the point. It may turn out that some aspects of psychic life fit conventional constructive models that do not reconcile with realism and vice versa; it may even be that theoretical standpoints that both enactivism and ecological psychology vehemently reject – for example, cognition as discrete symbol manipulation – are needed to account for dimensions of psychic life. This suggests an additional lesson, and one emphatically advanced by pragmatists: that explanations likely need to be pluralistic (see James, 1880/1992). Scientists have not unified physics, and the challenges posed here are inestimably less than those raised by the enormously complicated phenomenon of psychic existence.

A simultaneous problem and strength of academic work is the fervor with which proponents commit to specified views and the fact that critics only tend to read a small subsection of the literature they are attacking (Baggs and Chemero, 2020). A troubling and related tendency is that founding documents are too often taken as canonical when they are more accurately works in progress, steps in the right direction. “The Ten Commandments of Ecological Psychology” has already been written. It is a little tongue-and-cheek, and its authors acknowledge that it may add to already existing impressions that Gibsonians are fanatical, as opposed to conservative yet open minded researchers. However, the authors also write that “just as observant Jews and Christians ought not pick and choose which Commandments they follow, advocates of ecological psychology (or of genuinely embedded and embodied cognitive science) should see our commandments as a package deal” (Michaels and Palatinus, 2014, p. 19). While ecological psychology arguably is zealous, it is remarkable how much one can explain, for example, from the environment side without any recourse to the inner constitution of organisms. It is doubtful that ecological psychologists would have arrived at this productive point absent quasi-religious prohibitions against discussions of internal representations and other concepts that are part and parcel to mainstream cognitive science.

At the same time and at risk of offending both enactivists and ecological psychologists, it seems that unnecessary attempts to differentiate themselves from competitors and predecessors are at the root of some of today’s disputes. Gibson, (1979, pp. 138–140) for example, acknowledges debts to Gestalt theorists, while criticizing their distinction between the behavioral and geographical world as a pernicious subject-object dichotomy. This is in spite of the fact that it is close to the phenomenological distinction between the lived-world and second-order abstractions from it (see Crippen, 2015), which does not represent a subject-object divide.^4^ Varela et al. (1991), in their turn, do to Gibson what he did to Gestalt psychologists: they acknowledge a kinship, but then aggressively stress a radical departure, as opposed to simply framing their work as building on older models, and developing them in new directions.

What I have tried to do in this article is to highlight some unnecessary distinctions that enactivists and ecological psychologists insert, treating them as insurmountable differences. I have thereby attempted to affirm mutually reinforcing aspects of both schools, suggesting future directions for how they may combine into more encompassing accounts of embodied existence.

## Author Contributions

The author confirms being the sole contributor of this work and has approved it for publication.

### Conflict of Interest

The author declares that the research was conducted in the absence of any commercial or financial relationships that could be construed as a potential conflict of interest.

## Footnotes

^1^Michael Beaton (2016) offers a worthwhile account of enactive realism, but his arguments are not especially relevant to mine. Hence, while acknowledging his work, I do not integrate it into this article.

^2^Though I obviously think my account of enactivism is defensible, see Heft (2020) and Read and Szokolszky (2020) for excellent contrasting views.

^3^Some may object that eating only exploits properties that are already afforded and does not engender them, yet it then may be asked where exactly the line is drawn between exploiting and engendering. Do we say, for example, that seawater already affords cutting since trace elements in it can be processed into steel and then a knife?

^4^For similar argument, see Kiverstein et al. (2019).
